# Alzheimer's disease (AD) as a disorder of the plasma membrane

**DOI:** 10.3389/fphys.2013.00024

**Published:** 2013-02-15

**Authors:** Walter J. Lukiw

**Affiliations:** LSU Neuroscience Center, Louisiana State University Health Sciences CenterNew Orleans, LA, USA

Alzheimer's disease (AD) is a common, progressive degeneration of human brain structure and function, resulting in a deterioration of mood, behavior, functional ability, cognition, and memory (Alzheimer et al., 1995). Globally, 5 million new cases of AD are diagnosed annually, with one new AD case being reported every 7 s (Alzheimer Association, 2012). In this country taking care of AD patients places a tremendous socioeconomic burden not only on unpaid caregivers but on our health care system as a whole. The neuropathology of AD is highly variable and complex in its presentation, and the greatest risk factor for AD is age.

Besides the appearance of neurofibrillary tangles, at the root of the AD problem appears to be an up-regulation in the generation of small, toxic, and highly amyloidogenic 42 amino acid amyloid beta (Aβ42) peptides that self-associate, ultimately clumping into pro-inflammatory and microglia-activating senile plaques (SP). Interestingly, all of the enzymatic machinery responsible for the generation of Aβ42, and subsequent SP formation, are plasma membrane-resident secretases or modifier/accelerator proteins that are involved in the catabolic processing of the membrane-bound beta-amyloid precursor protein (βAPP). Besides the secretases, these modifier/accelerator accessory proteins include nicastrin, APH-1, presenilin-1, presenilin-2, sortilin, the TSPAN membrane proteins, and others; Table 1). Interestingly, dietary and systemic factors such as cholesterol, which perturb the biophysical structure of the phospholipid membrane and reorganize lipid raft domains where βAPP processing appears to occur, further contributes, via protein-lipid and protein-protein interactions, to membrane-mediated dysfunction of homeostatic βAPP neurobiology (Hicks et al., 2012; Kania et al., 2012). Indeed lipid raft nanodomains, of which there are millions in a single cell, have recently gained considerable attention as these membrane-embedded clusters of phospholipid-, sphingolipid- and cholesterol-enriched, integral and peripheral membrane proteins (such as the β - and γ-secretases) are instrumental in the processing of βAPP holoprotein and hence the amyloidogenic process itself (Hicks et al., 2012; Kania et al., 2012; Kosicek and Hecimovic, 2013; Table 1).

**Table 1:** **Role of the plasma membrane in the pathophysiology of AD**.

**Plasma membrane associated molecule or biological assembly**	**Plasma membrane association**	**Function/dysfunction**	**References**
βAPP	Integral	Intercellular signaling; source of all Aβ peptides and peptide fragments; unknown functions.	Lukiw, 2012a,b,c; Kosicek and Hecimovic, 2013
α-secretase (ADAM proteins)	Peripheral	Non-amyloidogenic βAPP processing	Hicks et al., 2012; Lukiw, 2012a,b,c; Kosicek and Hecimovic, 2013
β-secretase	Peripheral	Amyloidogenic βAPP processing	Hicks et al., 2012; Lukiw, 2012a,b,c; Kosicek and Hecimovic, 2013
γ-secretase	Integral	Amyloidogenic βAPP processing	Hicks et al., 2012; Lukiw, 2012a,b,c; Kosicek and Hecimovic, 2013
TSPAN	Integral	Promote non-amyloidogenic βAPP processing	Xu et al., 2009; Lukiw et al., 2012
TREM2	Integral	Innate immune surveillance; phagocytosis?	Niemitz, 2012; Singaraja, 2013
Exosomes	Plasma membrane derived	Paracrine communication; AD spreading (?); intercellular transport of genetic material (?)	Alexandrov et al., 2012; Lukiw et al., 2012; Fais et al., 2013
Lipid rafts	A dedicated “nanoregion” of the plasma membrane intrinsic to amyloid processing and amyloiogenesis	Cholesterol and other dietary lipids appear to modulate the processing of βAPP and amyloidogenesis	Hicks et al., 2012; Kania et al., 2012; Lukiw, 2012a,b,c; Orth and Bellosta, 2012; Kosicek and Hecimovic, 2013

Membrane-integral or membrane-peripheral associated modulators of Aβ42 peptide generation such as TSPAN12 or sortilin further contribute to the kinetics of formation, cleavage, processing, and speciation of βAPP (Lukiw, 2012a,b,c; Pallesen and Vaegter, 2012; Table 1). More recently, the participation of a membrane-spanning triggering receptor expressed in myeloid cells 2 (TREM2) protein supports a role for yet another plasma membrane-integral glycoprotein in phagocytosis and the clearance of Aβ42 peptides before they aggregate into SP (Niemitz, 2012; Singaraja, 2013). Of further interest is that plasma membrane-derived phospholipids and esterified dococahexaenoic acid (DHA) are the substrate for phospholipases, and hence the precursors for arachidonic acid cycle metabolites and cyclooxygenase conversion, that supports inflammatory signaling in the CNS (Heneka et al., 2010). Plasma membranes can also provide free DHA for conversion via a 15-lipoxygenase (15-LOX) into neuroprotectin D1 (NPD1), a potent neurotrophic docosanoid (Lukiw and Bazan, 2010). Hence, depending on the processing pathways and biological signals utilized, the plasma membrane can be the source of both beneficial and detrimental signals to further modulate amyloidogenic, inflammatory or neurotrophic aspects of the AD process.

Lastly, plasma membrane-derived exosomes are 30–90 nm diameter vesicles secreted into the extracellular milieu (Alexandrov et al., 2012; Lukiw et al., 2012; Fais et al., 2013). Besides containing various proteins and molecular constituents reflective of their cells of origin, these vesicles contain microRNAs as their most abundant nucleic acids (Alexandrov et al., 2012; Lukiw, 2012c). It is intriguing that these plasma membrane derived organelles may be capable of the *paracrine transfer of genetic information between cells*, either within the local environment of the brain or throughout the entire cerebrospinal or systemic circulation (Kania et al., 2012; Lukiw, 2012b,c; Lukiw et al., 2012; Fais et al., 2013). As exosome formation and release is mediated by the plasma membrane it is interesting to speculate that the microRNA-mediated transfer of genetic material between cells of the CNS and the intercellular transport of microRNA may actually be at least in part dependent on plasma membrane-mediated biological mechanisms. Again, environmental and dietary factors which modulate plasma membrane integrity, flexibility and lipid raft effects might not only be relevant in amyloidogenesis but also in paracrine microRNA trafficking and *the intercellular spreading of these soluble and mobile genetic signals*. Such activities may have profound importance in both health and disease. For example, plasma membrane biophysics, dynamics, and lipid raft domain perturbation by cholesterol and the HMG-CoA reductase inhibitors known as statins which target cholesterol metabolism, for example, might not only have effects on cholesterol incorporation into membranes and lipid raft formation but also in exocytosis and the potential of intercellular transfer of genetic information between cells (Hicks et al., 2012; Kania et al., 2012; Lukiw, 2012a,b,c; Fais et al., 2013; Kosicek and Hecimovic, 2013).

The interactions amongst these biological players with the plasma membrane remains poorly understood and require additional study. To cite just one further example is the potential involvement of neurotropic viral infection with AD, involving processes that are plasma membrane-mediated, pro-inflammatory, and evasive of the brain's innate immune response (Lukiw and Bazan, 2010; Ball et al., 2012). The papers provided in the current “*Frontiers Physiology”* issue entitled “*Membrane alterations and Alzheimer*'*s disease*” should certainly shed some light on these recently recognized plasma-membrane mediated events and how they impact both AD initiation and proliferation, and the AD process itself. Identifying their mechanisms, how they work and interact should yield a multitude of novel therapeutic strategies and targets that have not yet been considered for the clinical management of this tragic human neurological disorder.
